# Tumor growth inhibition and immune system activation following treatment with thorium-227 conjugates and PD-1 check-point inhibition in the MC-38 murine model

**DOI:** 10.3389/fmed.2022.1033303

**Published:** 2022-11-15

**Authors:** Axel Berg-Larsen, Anne Mobergslien, Ingrid Moen, Gebregziabher Petros, Alexander Kristian, Kristine Sponheim Gunvaldsen, Véronique Cruciani, Katrine Wickstroem, Roger Malerbakken Bjerke, Jenny Karlsson, Alan Cuthbertson

**Affiliations:** Bayer AS, Oslo, Norway

**Keywords:** thorium, alpha-therapy, PD-1/L1, radiotherapy, immune activation, immune checkpoint inhibitors, conjugate

## Abstract

Targeted thorium-227 conjugates comprise the combination of a monoclonal antibody with specificity for a tumor cell antigen and a 3,2-HOPO chelator enabling complexation of thorium-227 (Th-227). The radiolabeled conjugate functions as an effective delivery system of alpha-particle radiation to the surface of the tumor cell inducing difficult to repair complex DNA damage and cell death. In addition, the mechanism of action of targeted alpha therapy (TAT) appears to involve a significant component linked to stimulation of the immune system. We report herein evidence of immune activation and long-lasting immune protection of a TAT in a syngeneic model using the MC-38 murine cell line. Firstly, MC-38 cells were irradiated *ex vivo* with the thorium labeled antibody before subcutaneous implantation into mice. These mice were then rechallenged with MC-38 cells contra-laterally. In the group receiving irradiated cells, 9 out of 10 animals had no measurable tumor growth compared to aggressive tumor growth in the control group. Secondly, in an efficacy study, 500 kBq/kg of thorium labeled antibody alone or in combination with PD-1 checkpoint inhibitor gave statistically significant tumor growth inhibition compared to vehicle control. Animals with no measurable tumors were once again rechallenged contra-laterally with MC-38 cells. The re-growth of tumors was significantly delayed (approx. 60 days) in the treatment group compared to age-matched controls (approx. 30 days) in the monotherapy group. Interestingly, in the TAT/ PD-1 combination group no re-growth was observed demonstrating the potential of combining a TAT with checkpoint inhibition therapy. Finally, tumors were excised from treated mice and analyzed by flow cytometry and immunohistochemistry (IHC). Analysis revealed significant infiltration of CD8+ T-cells and mature dendritic cells compared to vehicle controls. Together these results indicated that an ongoing immune response from treatment with alpha radiation could be enhanced by check-point inhibition.

## Introduction

Targeted alpha therapy (TAT) is an emerging modality in the field of anti-cancer therapy and is dependent on the targeted delivery of alpha-particle emitting radionuclides to the tumor tissue (1). Targeted thorium-227 conjugates (TTCs) represent one such therapeutic approach comprising the combination of an antibody, with specificity for a tumor cell antigen, conjugated to a 3,2-HOPO chelator enabling complexation of Th-227 (2, 3). The resulting radiolabeled conjugate functions as an effective delivery system of alpha radiation inducing double strand DNA breaks and tumor cell death (4). In addition, due to the short range of the alpha-particle track there is limited damage to the surrounding healthy tissue (5).

The half-life of Th-227 (18.7 days) is compatible with the blood half-life of antibodies in humans, a key factor which allows for significant accumulation of the TTC in the tumor. Th-227 decay results in the generation of a total of five high-energy alpha and two beta particles ending with the stable element lead-207 (Pb-207) (6). The first daughter of thorium-227 is radium-223 (Ra-223), which also has a long decay half-life of 10.4 days. Radium-223 dichloride, the only TAT product currently approved by the FDA, is a calcium mimetic which deposits at the sites of abnormal re-modeling in bone metastases in patients with mCRPC. Adjacent tumor cells are therefore destroyed by crossfire of alpha particles (7). TTCs target the surface of the tumor cell and are not dependent on internalization as the path length of an alpha particle is between 2 and 10 cell diameters, extending the breadth of tumor specific antigens to non-internalizing targets. The high and localized energy deposited by the alpha-particle induces difficult to repair DNA double strand breaks in the target cell and TATs may therefore effectively evade many of the pathways by which cancer cells acquire resistance (8).

We have previously published TTCs with potential for the treatment of acute myeloid leukemia, renal-, breast-, lung cancer as well as mesothelioma and prostate cancer (9–14). We have also demonstrated their efficacy in *in vitro* and *in vivo* models as monotherapies as well as strong synergistic effects observed in combination with inhibitors of DNA damage repair (9, 11, 12). However, the influence TAT has on the immune system seems also to make an important contribution to the mechanism of action (15). The evaluation of synergies with immunotherapies have not been exhaustively studied and still need to be better understood (16).

The immune system plays a key role in inhibiting tumor growth through a process often referred to as immunoediting (17). Immune activation works in concert with selected anti-cancer treatments through the induction of immunogenic cell death further enhancing the therapeutic effect, the nature and potency of which, is dependent on the mechanism of action and tumor biology (18). This concept has been explored thoroughly in mice and has also been shown to be transferable to the human setting. The type and location of immune cell infiltrates in the tumor determines the outcome of immunoediting and is influenced both by the tumor environment and the choice of therapy (19). Immune cells can either infiltrate the core of the tumor or remain confined to the tumor margins and adjacent lymphatic vessels. Notably, infiltration post therapy can often be highly heterogeneous across specific tumor-types in a given population as well as in the metastatic setting within individual patients (20).

Depending on the type of immune response that is prevalent in the tumor, different outcomes are expected. In particular, the nature of the infiltrating T-lymphocytes (CD3+ T-cells) is critical for a positive outcome. An immune response dominated by T_*H*_1 helper T-cells and CD8+ cytotoxic T-cells is often correlated with inhibition of tumor growth and complete tumor regression both in mice and humans (21), while a response dominated by T-regulatory cells (FOXP3+) is often neutral or even negatively correlated with patient overall survival (22). Likewise, the nature and type of the innate immune cells in the tumor may have a profound role to play. A tumor with a large macrophage population is correlated with poorer outcomes than one where a large mature dendritic cell population is present (23).

The success of check-point inhibitors has led to a greater understanding of the nature of the immune response even in advanced tumors (24). Programmed death-ligand 1 (PD-L1) and its receptor PD-1 on immune cells have been the most frequent targets of such therapy (25). PD-L1 is normally not expressed at high levels in tissue that is not immune-privileged in some manner, but is upregulated in response to certain inflammatory cytokines such as interferon-γ (IFN-γ) (26). The PD-L1/PD-1 interaction deactivates T-cells and prevents target cell killing, and this mechanism is used by tumor cells to avoid immunoediting. As such, there is often a latent immune response against even advanced cancers already present at the tumor site, being suppressed by such check-points as well as other immune system avoidance strategies (27). By introducing check-point inhibitors it is possible to reactivate the immune response and induce regression of tumor growth, but it is still the case that only a minority of patients respond to a significant degree. Check-point inhibition can therefore be administered in combination with other therapies to further enhance efficacy and patient outcomes (28).

We describe herein the effect of a combination of TAT and a check-point inhibitor in murine models of CRC utilizing the tumor cell line MC-38 in an attempt to explore potential synergies with TAT (29). The principle aim of this study was to investigate if such an effect can be observed using a PD-L1 TTC in combination with PD-1 targeted therapy.

## Materials and methods

### Preparation of thorium conjugated antibodies

Conjugates were prepared as described in Hagemann et al. (9), using a recombinant murine anti-PD-L1 antibody based on the sequence of atezolizumab to make murine IgG1 (Anti-PD-L1 mIgG1 Kappa, RG7446 chimera, PPB-6390), produced in-house by Bayer AG (Wuppertal, Germany). For isotype antibody conjugate, mouse isotype BAY 2862727 (SSB-Isotype-mIgG1Lambda) was used. HOPO-chelator-to-antibody ratio was measured using SE-HPLC at 280 nm (antibody) and 335 nm (HOPO). Briefly, the conjugates were prepared by coupling an *N*-hydroxysuccinimide-activated 3,2-hydroxypyridinone (HOPO) chelator covalently to the ϵ-amino groups of the lysine residues. The chelator-to-antibody ratio (CAR) for the prepared batch was 0.6. Radiolabeling with thorium-227 was done in 30 mmol/L citrate, 70 mmol/L NaCl, 0.5 mg/mL PABA, 2 mmol/L EDTA, pH 5.5 at room temperature for 1 h.

### Media and cell lines

As a target cell for TTC treatment we used the colon cell line MC-38 (NMI) cultured in RPMI (Biowest) + 10%FBS (Hyclone) 1%P/S (Corning) + 1xMEM-NEAA (Gibco) + 1xNaPyr (Gibco) + 0.5 μg/ml Blasticidin (Life Technologies). As a control cell line, we used the skin cell line B16-F10 (ATCC) cultured in DMEM/Ham’s F12 (Biochrom.) + 10% FBS (HyClone) + 1% P/S (Corning).

Cell viability was measured using CellTiter Glo (# G924C, Nerliens Meszansky) read of on an EndoSafe platereader after 6 days of TTC or control treatment.

### *In vivo* procedures

The *in vivo* procedures described in this manuscript have all been approved by the National Animal Research Authority and were carried out in compliance with the European Convention for the Protection of Vertebrates Used for Scientific Purposes. Female C57BL/6JRj C57B/k (H-2b) aged 4, 5, or 16 weeks, weighing 15–18 or 18–25 g, acquired from Janvier Labs, France were used as host animals. 200.000 MC-38 cells were inoculated subcutaneously (s.c.) in the right flank for efficacy and distribution studies, 100.000 MC-38 or B16-F10 cells were inoculated (s.c.) in the left flank for rechallenge experiments.

For distribution experiments, PD-L1-TTC or an untargeted isotype Th-227 conjugate) was administered at 0.14 mg/kg and 500 kBq/kg via the tail vein. Organs were harvested at predefined timepoints, and the distribution was investigated by measuring thorium-227 in selected organs by high purity geranium detector detector (HPGe). Pre-treatment with 0.2 mg/animal anti-PD-L1 antibody was given to separate groups by intraperitoneal (i.p.) injection the day before TTC treatment. In the efficacy and rechallenge experiments, animals were treated with PD-L1-TTC or isotype conjugate at 0.14 mg/kg and 500 kBq/kg, with or without 0.2 mg/animal anti-PD-L1 or anti-PD-1 antibody pretreatment. Anti-PD-L1 was identical to non-conjugated PD-L1 as described above. Anti-PD-1 antibody was supplied by BioXCell (RMP1-14). For each rechallenge with MC-38 or B16-F10 cells, five age-matched control animals received the same inoculation of cells.

When pre-inoculation irradiation with thorium-227 was required, cells were exposed to 40 kBq/ml thorium-227 for 48 h. Excess thorium-227 was removed by wash cycle and cells were harvested and inoculated as described above.

### Isolation of immune cells from mice tumor and spleen

Tumors were cut into small pieces and dissociated using a tumor dissociation kit (Miltenyi Biotec) with the gentleMACS tumor dissociator according to manufactures protocol. CD45+ cells were isolated from the resulting cell suspension using magnetic microbeads (Miltenyi Biotec) on a MACS separator column (Miltenyi Biotec) mounted on a magnetic scaffold.

Spleens were mashed through a 70 μm nylon cell strainer using a sterile plunger. The resulting spleenocytes were washed in PBS and then resuspended in 5X RCB lysis buffer (Milenyi Biotec). Once erythrocytes were removed, the cells were strained through a 40 μm nylon filter and washed in PBS three times in succession for a final suspension of spleenocytes.

### Cell toxicity assay

MC-38 cells were seeded in 384 wells plates and incubated over night at 37°C, 5% CO_2_. Next day, added 0.03% tween to the PD-L1 TTCs and isotype Th-227 conjugate before they were added to the cells, by using the D300e digital dispenser with concentrations as indicated. The plates were normalized to highest fluidic volume.

After 7 days incubation was 30 μl CellTiter-Glo added to each well. Read-out was luminescence on Wallace Envision Platereader.

### γH2AX and DAMP measurement

MC-38 cells were seeded in six wells plates, 250,000 cells per well in 5 ml medium. Next day, added the compounds; 2, 10, and 25 kBq/ml was used for the PD-L1-TTCs and 10 and 25 kBq/ml for isotype Th-227 conjugate. After 48 and 72 h exposure the cells were harvested, permeabilized and stained with yH2AX or DAMPS markers as described below.

### Flow cytometry

Cells were stained for flow cytometry using CD3, CD4, and CD8 antibodies as well as dead cell marker for T-cells, and CD86, CD64, and CD11c for dendritic cells and macrophages. T-regulatory cells were fixed and stained using the T reg detection kit Miltenyi Biotec) according to manufacturer’s specification.

Antibodies used for flow were as follows: α-CD4 PE (Miltenyi Biotec), α-CD8 Vivobright FITC (Miltenyi Biotec), α-CD3 APC (MIltenyi Biotec), α-CD11c FITC (MiItenyi Biotec), α-CD86-PE (Miltenyi Biotec), and α-CD64-APC (Miltenyi Biotec).

Danger-Associated Molecular Patterns were analyzed by staining targeted cells with anti-human Calreticulin (CRT)-A647 (Bioss), anti-human HSP70-A647 (Bioss), anti-human GRP94-A647 (Bioss), and anti-human HMGB1-A647 (Bioss). yH2AX was analyzed by staining the cells using anti-human yH2AX (Biolegend). All antibodies were cross-reactive with mice.

To measure immunomarkers, the cells were stained with APC anti-mouse IFNAR-1 (Biolegend), APC anti-mouse CD252 (Biolegend), APC anti-mouse CD54 (Biolegend), APC anti-mouse CD152 (Biolegend), APC anti-mouse CD274 (Biolegend), APC anti-mouse CD137 (Biolegend), APC anti-mouse CEACAM-1/CD66a (R&D), Alexa Fluor 647 anti-mouse B7-H2 (R&D) Alexa Fluor 647 anti-mouse TRAILR1/TNFRSF10A (Novus), Alexa Fluor 647 anti-mouse CD27 ligand/TNFSF7/CD70 (Novus).

All cells were analyzed on a Guava Easycyte 8HT flow cytometer.

### Immunohistochemistry

Immunostaining of tumor sections was performed on 3 μm-thick FFPE (formalin fixed paraffin embedded) sections. Sections incubated in 60°C for 30 min in a heating cabinet before deparaffinization in xylene and then hydrated in graded alcohol solutions. Target retrieval performed in a preheated steamer for 25 min in Target Retrieval solution pH9, 10x (Dako). Thereafter cooled on ice until temperature reached 50°C. Slides were placed in a humidifying chamber and incubated with primary Ab for 60 min. Sections were stained with primary Ab against PD-L1 (Nordic Biosite, 1:100 dilution), CD4 (AbCam, 1:1,000 dilution) and CD8 (AbCam, 1:2,000 dilution). Slides then blocked with 3% peroxidase blocking solution, H_2_O_2_ (Nordic Biosite), before incubation with Envision labeled polymer anti Rabbit (Dako) for 30 min. Antibody-antigen complex was visualized with DAB substrate system for 10 min (Dako) followed by counterstaining with Hematoxilin (Sigma) for 60 s. Slides were dehydrated in increasing alcohol-containing solutions, ending in xylene, before mounting. Positive control tissue added to all staining set-ups (sections of spleen and lymph with a known expression of the target).

### Statistical analysis

Performed as indicated in figure legends. Differences between groups were considered statistical significant when *p* < 0.05.

## Results

### *In-vitro* targeted thorium-227 conjugates treatment induces danger associate molecular patterns upregulation and immunogenic cell death

**FIGURE 1:**
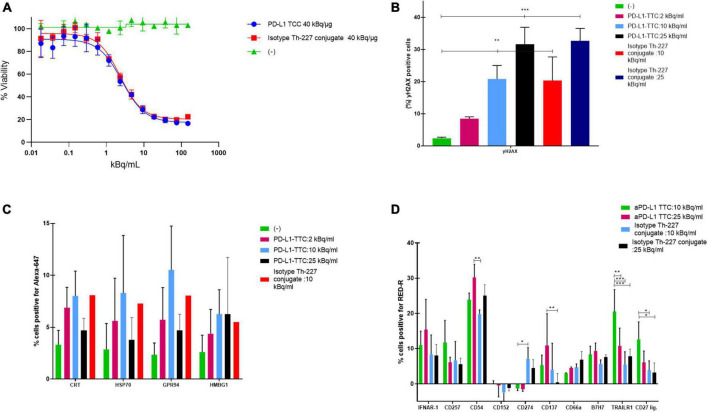
**(A)** Cell viability of MC-38 treated for 7 days with indicated targeted thorium-227 conjugates (TTC) or cold conjugate. **(B)** MC-38 cells (%) positive for yH2AX by flow cytometry staining after 7 days of treatment with indicated TTC or cold conjugate. **(C)** MC-38 cells (%) positive for four common DAMPs by flow cytometry after 7 days of treatment with indicated TTC or cold conjugate. **(D)** MC-38 cells (%) positive for 10 additional DAMPs by flow cytometry after 7 days of treatment with indicated TTC or cold conjugate. Tukey’s multiple comparison test, *p < 0.05, **p < 0.01, ***p < 0.001.

To determine the effectiveness of the radiolabeled conjugate we first measured the response of our target cells *in vitro*. PD-L1 TTC was cytotoxic to MC-38 cells (Figure 1A), and DNA damage measured by detecting γH2Ax levels by flowcytometry (Figure 1B). In culture, a dose dependent increase in the danger associate molecular patterns (DAMPS) calreticulin, HSP70, GPR94, and HMBG1, expressed on the cell surface, was observed (Figure 1C) indicating the potential for induction of immunogenic cell death (ICD) by alpha radiation (30). This effect was also significant for the isotype Th-227 conjugate. We also observed a strong dose dependent expression of immune marker ICAM-1 (CD54) and cytokine receptors IFNAR-1 and CD137 (Figure 1D).

**FIGURE 2:**
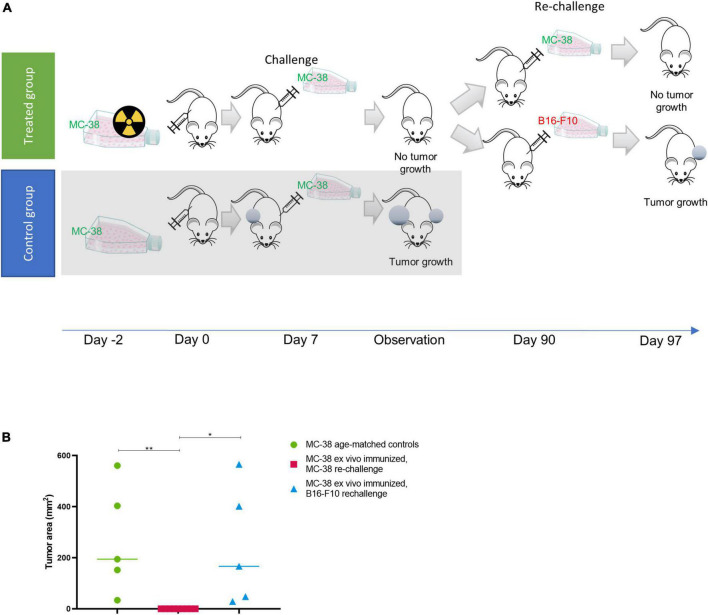
*Ex vivo* immunization with irradiated MC-38 cells. **(A)** Scheme detailing *ex-vivo* immunization using targeted thorium-227 conjugates (TTC) irradiated MC-38 cells. In brief, mice were implanted with MC-38 cells pretreated with alpha radiation (*n* = 10), or with healthy MC-38 cells to serve as control (*n* = 5). The mice were then challenged with a new MC-38 implantation after 7 days, and the mice that did not develop tumor were rechallenged once again after 90 days using MC-38 or the unrelated B16-F10 cell line (*n* = 5). **(B)** Size of tumors at day of sacrifice in each treatment group. 0 indicates no measurable tumor observed at sacrifice. Student’s *T*-test, *p < 0.05 and **p < 0.01.

To investigate whether such an immune response could induce immunity *in vivo*, we performed a study similar to Gorin et al. (31) whereby we firstly irradiated MC-38 cells with thorium conjugated antibodies *in vitro* 48 h prior to subcutaneous implantation of the irradiated cells in mice. After 7 days mice were challenged with living, non-irradiated cells (Figure 2A).

In the group initially receiving irradiated MC-38 cells, 9 out of 10 mice rejected the non-irradiated MC-38 tumor at challenge (day 7), while growth was observed in all mice in the age-matched control group (Figure 2A). The specificity for MC-38 cells was evaluated by rechallenge of the immunized mice, with either B16-F10 or MC-38 cells 90 days after the initial cell inoculation (Figure 2B). Tumor growth was observed in 5 out of 5 mice rechallenged with B16-F10 cells, while the mice rechallenged with MC-38 showed no tumor growth, indicating that the immunization effect was specific for MC-38 cells.

### Programmed death-ligand 1 targeted thorium-227 conjugates monotherapy and combination with anti-PD-1 check-point inhibition

**FIGURE 3:**
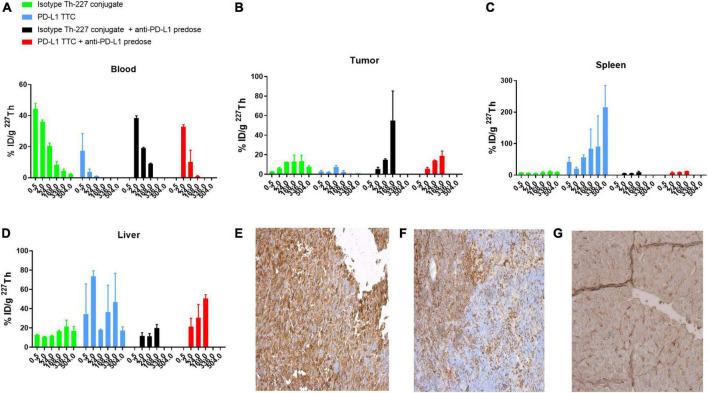
**(A–D)** Biodistribution of 500 kBq/kg isotype Th-227 conjugate or PD-L1-TTC in MC-38_EGFP mice without and with 0.2 mg anti-PD-L1 predosing before dosing. *n* = 3/group/timepoint. **(E)** PD-L1 expression in MC-38 tumor. **(F)** PD-L1 expression in C57BL/6 spleen. **(G)** PD-L1 expression in C57BL/6 liver. For blanks of IHC staining, see Supplementary Figure 4.

Based on the initial results, we then explored whether the PD-L1 TTC would be efficacious *in vivo*, inducing a similar immunization effect following systemic administration. A biodistribution study in the MC-38 model initially demonstrated rapid blood PK (Figure 3A) for the PD-L1 TTC due to the rapid accumulation in the spleen with an observed uptake of >50% ID/g after 24 h (Figure 3C).

As a result, tumor accumulation was low at around 5–8% ID/g at 24 h, decreasing to 2–3% ID/g at the 168 h timepoint (Figure 3B). The high splenic uptake was reduced by blocking with a pre-dose of PD-L1 (i.p. 0.2 mg/kg) resulting in an increased tumor uptake of the PD-L1 TTC to 20% ID/g at 168 h.

The high level of PD-L1 expression in both liver and spleen was confirmed by IHC staining (Figures 3F,G) with significantly less staining observed in the tumor (Figure 3E). As such, pre-dosing of the check-point inhibitor appeared to prevent excessive binding of the PD-L1 TTC in liver and spleen allowing for improved delivery to the tumor.

**FIGURE 4:**
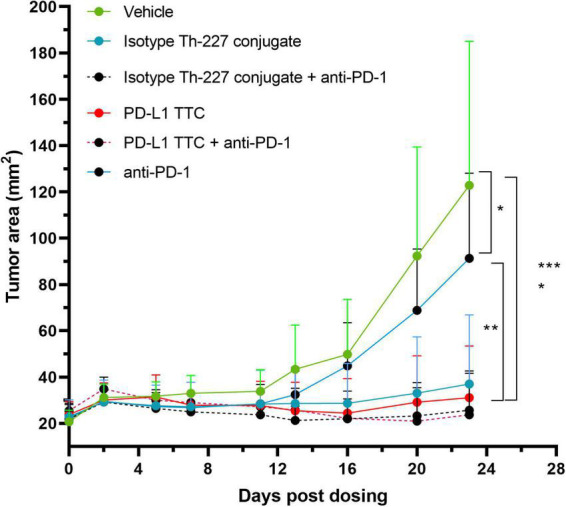
MC-38 tumor bearing mice were treated with vehicle, 500 kBq/kg isotype Th-227 conjugate, 500 kBq/kg PD-L1-TTC, anti-PD-1 (10 mg/kg, 2× weekly) or a combination of 500 kBq/kg PD-L1-TTC and anti-PD-1 (10 mg/kg, 2× weekly). All mice were pre-dosed with 0.2 mg of anti-PD-L1 prior to TTC dosing. After 23 days all TTC treatment groups demonstrated statistically significant tumor growth inhibition compared to the vehicle control group, (*n* = 8) as analyzed by 2-way ANOVA with Tukey’s post hoc test *=0.05, **=0.01, ****=0,0001.

An efficacy study was then performed with an anti-PD-1 check point inhibitor alone or in combination with isotype Th-277 conjugate or PD-L1 TTC. While no effect on tumor growth was observed for the anti-PD-1 group alone, statistically significant inhibition of tumor growth was observed for all other treatment groups including the isotype Th-227 conjugate dosed at 500 kBq/kg with no apparent synergy for either PD-L1 TTC or isotype Th-227 conjugate with addition of the check point inhibitor (Figure 4). On day 23 after treatment, there were six mice with no measurable tumor, all treated with TTC. Interestingly, two of these mice were treated with isotype conjugate, 1 with isotype conjugate + anti-PD-1, 1 with PD-L1 TTC and 2 with PD-L1 TTC + anti-PD-1, showing no clear bias between treatment groups.

### Mice rechallenged after treatment with combination therapy show no tumor re-growth

To understand if efficacy was related to the induction of an effective and specific immune response these animals were further challenged with a second inoculation of MC-38 and B16-F10 cells. The six responding mice were randomized into two treatment groups (*n* = 3) along with a third age-matched control group (*n* = 5). To allow for full immune recovery, the mice were left until day 129 before rechallenge.

**FIGURE 5:**
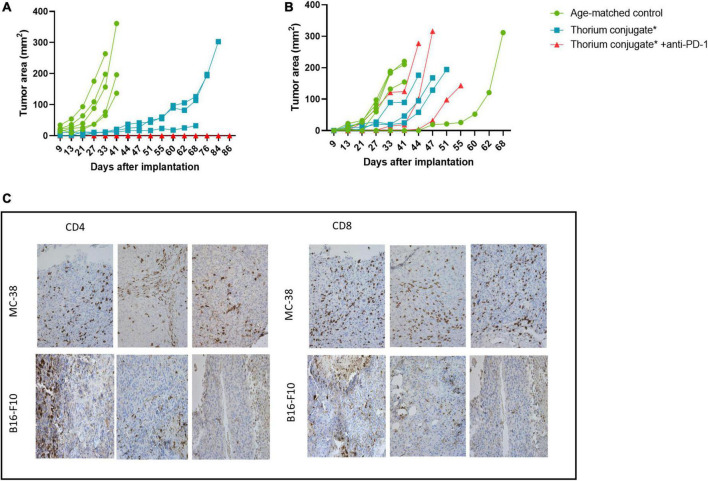
**(A)** Growth curve for rechallenge with MC-38 cells after treatment as indicated, *n* = 5 in control groups, three in treatment groups. **(B)** Growth curve for rechallenge with B16-F10 cells after treatment as indicated, *n* = 5 in control groups, three in treatment groups. **(C)** IHC slides from MC-38 and B16-F10 tumors stained for CD4+ and CD8+ T-cells. Slides are from regrowing tumors of monotherapy treated mice. *Animals previously treated with either PD-L1 TTC or isotype Th-227 conjugate.

In the age-matched control group the tumors grew rapidly over the first 30 days post implantation while in the TTC monotherapy group the growth was significantly delayed out to 60 days. Surprisingly, mice from the combination group showed no re-growth of MC-38 tumors (Figure 5A). To evaluate the specificity of the response all treated mice (*n* = 6) were inoculated with B16-F10 cells contralaterally 42 days after MC-38 cell rechallenge and compared once again to a new age-matched control group (*n* = 5). B16-F10 cells tumor growth was observed in all mice (Figure 5B) including the anti-PD1 combination groups.

The tumors which had regrown in the TTC monotherapy groups were excised and immunohistochemistry performed to investigate the extent of T-cell infiltration (Figure 5C). Both CD8+ and CD4+ T-cells were present in large numbers in the MC-38 tumors but not in the B16-F10 tumors. Based on these data, we wished to further investigate the infiltration of immune cells into treated tumors in a new study.

### Cytotoxic T-cells infiltrate the tumors and contribute to cancer treatment

**FIGURE 6:**
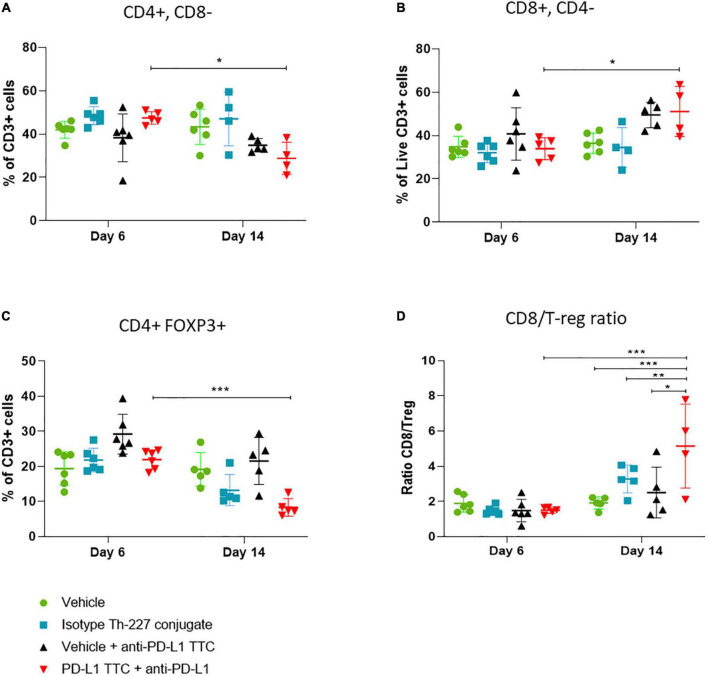
Lymphocytes (%) that are CD3+ in tumors, in each treatment group, *n* = 5/group. **(A)** CD3+ cells (%) that are CD4+ CD8- in tumors, representing T-helper cells, *n* = 5/group. **(B)** CD3+ cells (%) that are CD8+ CD4-, representing cytotoxic T-cells, *n* = 5. **(C)** CD3+ cells (%) that are CD4+ FOXP3+ CD25+ in tumors, representing T-regulatory cells, *n* = 5/group. **(D)** Ratio of CD8+ cells to T regulator cells in tumor, *n* = 5. Student’s *t*-test *p < 0.05, **p < 0.01, ***p < 0.001.

In this follow-up study all mice were pre-dosed with anti-PD-L1 antibody prior to PD-L1 TTC monotherapy and combination treatments. Tumors and spleens were isolated from all mice and immune cells extracted. Flow cytometry (Supplementary Figure 1) performed 6 days after treatment revealed CD3+ T-cell infiltration in all tumors (Figure 6). Over time we observed a change in the sub-population of T-cells from day 6 to day 14. There was a reduction of CD4+ cells (Figure 6A) in the TTC-treated group in comparison to the other groups, with a corresponding increase in CD8+ T-cells (Figure 6B). Identifying T-regulatory cells (Tregs) in the tumors as FOXP3+ CD4+, we also observed that Tregs are reduced significantly in PD-L1 TTC treated tumors, and slightly reduced in other treatment groups outside of vehicle control (Figure 6C). The ratio of Tregs to CD8^+^ cells (Figure 6D) was also significantly increased in the TTC+PD-L1 treatment group, indicating an ongoing cytotoxic immune response, correlating with the shrinking tumor size (Figure 3).

### Mature dendritic cells supporting immune responses in targeted thorium-227 conjugates treated tumors

**FIGURE 7:**
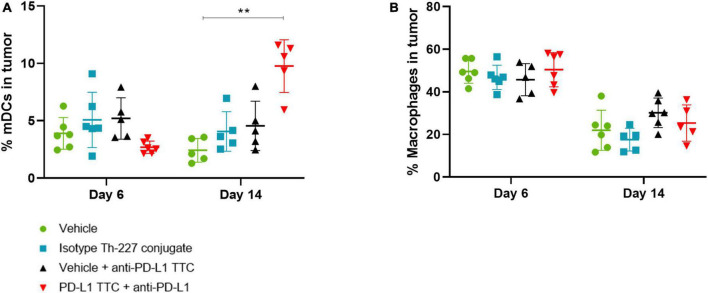
**(A)** Mature DCs [CD11c+, CD86+, CD64- (%)] in tumors in each treatment group, *n* = 6/group. A significant increase is seen after 14 days in the combination therapy group. **(B)** Macrophages [CD11c+, CD64+ (%)] in tumors in each treatment group, *n* = 6/group. Student’s *t*-test **p < 0.01.

In addition to T-cells we also isolated dendritic cells and macrophages from the tumors to investigate the innate immune response to the TTC treatment. We found that mature DCs (CD45+, CD11+, CD86+, and CD64-) were increased after 14 days in PD-L1 TTC treated tumors, but not in any other group (Figure 7A). Macrophages (CD45+, CD64+, and CD11-) were high after 6 days but decreased in all treatment groups after 14 days (Figure 7B). Together, these results indicate that dendritic cells are driving the innate immune response in the tumors in contrast to macrophages.

## Discussion

The cytotoxic effect of radiotherapy in general is well documented (32). However, it appears that direct cell death inducing apoptosis and necrosis is only partially responsible for the observed efficacy and that the immune response triggered by the radiation also plays a role to a significant degree (33–35). Radiation therefore functions as an immunological adjuvant, triggering an immune response that overcomes the tumors inherit acquired immune evasive state (36, 37). The combination of check-point inhibitors and high dose radiation in some indications has demonstrated synergy in several studies (38–41). The immunogenicity of cells targeted by radiation therapy varies from tumor to tumor depending on both type of radiation and the cell lines inherit immunogenicity (31). In the current study, we show that alpha radiation in combination with check-point inhibition not only induces tumor regression but also appears to lead to the increased infiltration of CD8+ T-cells into tumors and the development of long-lasting immunity against tumor cells.

Our initial goal was to show that TAT treatment alone could induce a sufficient immune response to allow for long lasting immunity, as has been previously published (15, 31). MC-38 is an immunogenic cell line that also expresses PD-L1 and as such is a good target for both check-point inhibition and TAT treatment (Figures 1A,B). The *in vitro* treatment with both PD-L1 TTC and isotype Th-227 conjugate induced equivalent amounts of cell death (Figure 1A) and upregulation of markers of immunologic cell death including the DAMPs HMGB1, CRT and HSPs (Figures 1C,D), known to have immunostimulatory effects in tumors (42–45). Although there is no known antigen for the isotype antibody expressed on the tumor cells, it appears there is some non-specific binding to the MC-38 cell line, indicating that the antibody is not a true negative control. It is also of note that MC-38 cells do not express much PD-L1 *in vitro* (data not shown) but upregulate PD-L1 *in vivo* due to the changing microenvironment (Figure 3). Thus the observed *in vitro* effects are due to the damage induced by alpha particles and not dependent on antibody specificity.

By irradiating target cells in vitro followed by inoculation into immune competent mice, an immune response was triggered that resulted in long lasting immunity against MC-38 cells (Figure 2A). Rechallenged with fresh MC-38 cells, tumor growth was completely inhibited in 9 out of 10 animals in the group of immune stimulated mice, but not in the age-matched control mice or in mice inoculated with the B16-F10 cell line (Figure 2B). This demonstrated that some degree of specific immune protection had been achieved comparable to the previously published work of Gorin et al. (31).

Having demonstrated that we could induce immunity in this manner, we then went on to explore the potential of the TAT inducing immune responses directly *in vivo*. MC-38 tumors express PD-L1 *in vivo* (Figure 3E), however, as PD-L1 is also expressed in normal tissues such as spleen and liver (Figures 3F,G) a PD-L1 pre-dose was required to reduce non-target organ uptake and increase tumor targeting (Figures 3C,D). Interestingly, the non-targeting isotype antibody also displayed relatively high tumor uptake, similar to PD-L1 TTC (Figure 3B), likely due to a combination of non-specific binding (Figure 1), and a significantly enhanced permeability and retention effect in this tumor model (46). The high tumor uptake of both TTCs may also explain why statistically significant tumor growth inhibition was achieved for both PD-L1 TTC and isotype Th-227 conjugate as a monotherapy and in combination with PD-1 check-point inhibition (Figure 4). Anti-PD-1 alone had only a modest effect in this tumor model (Figure 4). Alpha radiation treatment appears therefore to induce an immune response through immunogenic cell death, with enhanced efficacy in combination with check-point inhibition. This has been previously reported with other methods of irradiation in combination with check point inhibition (47).

A common response to immune activation within a tumor is the upregulation of checkpoint ligands including PD-L1 resulting in the loss of significant immune response (41, 48). Any increase in expression would be expected to increase the radiation dose delivered to the tumor by the PD-L1 TTC as more radionuclide is delivered to the target. Furthermore, the addition of an anti-PD-1 antibody seemed to complement alpha radiation treatment, likely due to the presence of PD-1 expressing CD8+ T-cells in the tumors (Figure 6B). The addition of anti-PD-1 would counteract increasing PD-L1 levels and work by inducing T-cell activation even as more cells express PD-L1. PD-1 is also known to be expressed at increasing levels during persistent encounters with antigens, as is likely in a tumor environment. The effect of the combination therapy should increase over time, as the immune response continues, and immune memory is generated. As immune memory is also known to be modulated through the PD-1 pathway, the effect of the combination therapy should also be persistent in rechallenge experiments (49), as we indeed observed (Figure 5).

In order to investigate further the key components of immune activation and protection, mice with no detectable tumor from the combination and monotherapy groups (Figure 4) were pooled and after 90 days rechallenged with MC-38 cells. Interestingly, Figure 5 shows that the age-matched control group all grew tumors of >100 mm^2^ in 30–40 days while in the TTC monotherapy group regrowth was significantly delayed with tumors >100 mm^2^ measured after 60 days. Surprisingly there were no measurable tumors in the group treated with the combination of TTC and PD-1. This was a clear indication that the combination therapy had triggered a stronger, more robust immune response leading to long term protection. To further characterize the immune response, tumors from the monotherapy group were excised on regrowth (>200 mm^2^) and cells extracted for analysis and IHC. IHC (Figure 5C) revealed the presence of infiltrating T-cells in the TTC treated group but not in the age-matched control tumors. This response was enough to delay tumor growth but not enough to result in long term protection potentially due to the development of tumor immune evasion or resistance (50, 51).

We postulated that the observed longer-term protection of the anti-PD-1/TTC combination therapy reflected the nature of the immune response. The combination group was less susceptible to escaping immune surveillance, allowing the development of a robust intra-tumoral memory effector T-cell response (52) which eliminated the nascent tumor. The specificity of the immune response was also confirmed by inoculation of the mice with B16-F10 (Figure 5B). B16-F10 tumors grew in both control animals and animals with complete tumor regression indicating that the immune response was specific for MC-38 cells.

In addition to IHC, we extracted infiltrating immune cells from TTC treated tumors. As expected, we found both CD4+ and CD8+ T-cells as well as Tregs (Figure 6) infiltrating the tumors to varying degrees, depending on time and treatment. In many tumor environments regulatory cells and CD4+ T-cells would dominate the response and produce an immune tolerant microenvironment (53). In addition, check point inhibition alone has the potential to increase immune evasion through proliferation of Tregs in the tumor when no additional immune stimulation is present (54). However, the combination of alpha therapy induced ICD and check-point inhibition appeared to allow for an increase in active infiltrating CD8+ T-cells (Figure 6B), the alpha radiation promoting an immune stimulatory environment that supports CD8+ T-cell activation and the anti-PD-L1 breaking tolerance from PD-L1 expressed on the tumor cells (55, 56). The result is a cytotoxic immune response dominated by CD8+ cells over Tregs as expressed in the high ratio of CD8+ to Tregs in the tumor (Figure 6C). This ratio has been linked to breaking Treg dependent resistance in tumors (51), preventing metastasis (57) as well as better prognosis for patients in ICI treatment (50, 58, 59). The increase in CD8+/Treg ratio in the combination group seems to confirm the previous findings and support the observations.

The presence of mature dendritic cells and macrophages in the tumor microenvironment has a large effect on the developing immune response and even the prognosis of tumor growth and metastasis (60–62). The presence of large amounts of mature dendritic cells is helpful for the immune response (62), while macrophages, especially M2 macrophages, are often immunosuppressive (61). In tumors treated with combination therapy, we see an increase in mature dendritic cells over time (Figure 7A), while we see a drop in macrophages in all treatment groups (Figure 7B). This supports our T-cell data, and the hypothesis of a developing immune response initially dominated by infiltrating macrophages and immature DCs, which over time shifts to mature DCs supporting CD8+ T-cells as effector cells leading to tumor elimination.

In conclusion, the persistent immune effect we observed is exemplified by the complete rejection of tumors implanted in mice with complete tumor regression from previous treatment. It is clear from the *ex vivo* immunization data (Figure 2) that this effect is mainly from the radiation induced killing of the target cells, but the presence of the check-point inhibitor also plays a crucial role for the treatment to work optimally *in vivo* (Figure 5). Intriguingly we observe a clear effect even in the mice where complete tumor regression was achieved initially but rechallenge with tumor resulted in new tumor growth. The original combination of TTC and ICI appears to have induced a more lasting immunity due to the nature of the initial response they produced together with a strong cytotoxic T-cell component being critical (63, 64). Although the conclusions from this study are based on modest sample size, it appears to provide promising evidence of immune stimulatory effects of TAT in combination with check-point inhibitors. MC-38 is already known to be an immunogenic cell line (15) shown to respond to immune check point inhibition (65). Based on these preclinical findings, combinations of TAT and ICI may be of interest in treatment of cancers with high mutational load in a similar manner. It remains to be seen if such combinations can be used to make nonimmunogenic tumors respond to check-point inhibition. In addition, these combinations may also have clinical relevance in indications were check-point inhibition is already approved by the FDA, such as melanoma and non-small cell lung cancer, in patient populations that become non-responsive to ICI.

## Data availability statement

The raw data supporting the conclusions of this article will be made available by the authors, without undue reservation.

## Ethics statement

The animal study was reviewed and approved by Norwegian Food and Safety Authority.

## Author contributions

AB-L, AC, RB, and JK conceived and designed the project. AB-L, AM, IM, VC, KW, KG, GP, and AK contributed data and performed the experiments. AB-L, AM, IM, JK, GP, and AC performed the analysis. AB-L and AC wrote the manuscript, with significant support from IM and JK. All authors contributed to the article and approved the submitted version.

## Supplementary material

Supplementary Figure 1 Gating scheme for all cells. **(A)** Gating scheme for T-cells in tumor. CD45+ cells from tumor were gated on FSC/SSC, then live vs. dead, then CD3+. CD3+ T-cells were gated as CD4+. CD8+ or CD4+ FOXP3+ as shown. **(B)** Gating scheme for dendritic cells and macrophages in tumor. CD45+ cells from tumor were gated on FSC/SSC, then live vs. dead, then CD11c+. Macrophages were gated as CD64+, mDCs as CD64- CD86. **(C)** Gating scheme for T-cells in spleen. CD45+ cells spleen was gated on FSC/SSC, then live vs. dead, then CD3+. CD3+ T-cells were gated as CD4+. CD8+ or CD4+ as shown. **(D)** Gating scheme for dendritic cells and macrophages in spleen. CD45+ cells from spleen were gated on FSC/SSC, then live vs. dead, then CD11c+. Macrophages were gated as CD64+, mDCs as CD64- CD86.

Supplementary Figure 2 **(A)** Lymphocytes (%) that are CD3+ in spleen, in each treatment group, *n* = 5/group. **(B)** CD3+ cells (%) that are CD4+ CD8- in spleen, *n* = 5/group. **(C)** CD3+ cells (%) that are CD8+ CD4- in spleen, *n* = 5/group. **(D)** Ratio of CD8+ cells to CD4+ in spleen, *n* = 5/group. Student’s *t*-test **p* < 0.05, ***p* < 0.01, ****p* < 0.001.

Supplementary Figure 3 **(A)** Mature DCs [CD11c+, CD86+, CD64-(%)] in spleen, in each treatment group, *n* = 5/group. **(B)** Macrophages [CD11c+, CD64+ (%)] in spleens, in each treatment group, *n* = 5/group. Student’s *t*-test **p* < 0.05.

Supplementary Figure 4 Blank IHC samples **(A)** Blank MC-38 tumor. **(B)** Blank C57BL/6 spleen. **(C)** Blank C57BL/6 liver.
