# Supplementary figures and images for: Tumor growth inhibition and immune system activation following treatment with thorium-227 conjugates and PD-1 check-point inhibition in the MC-38 murine model
Source: Front Med (Lausanne). 2022 Nov 15;9:1033303. doi: 10.3389/fmed.2022.1033303 (PMC9705341; doi:10.3389/fmed.2022.1033303)

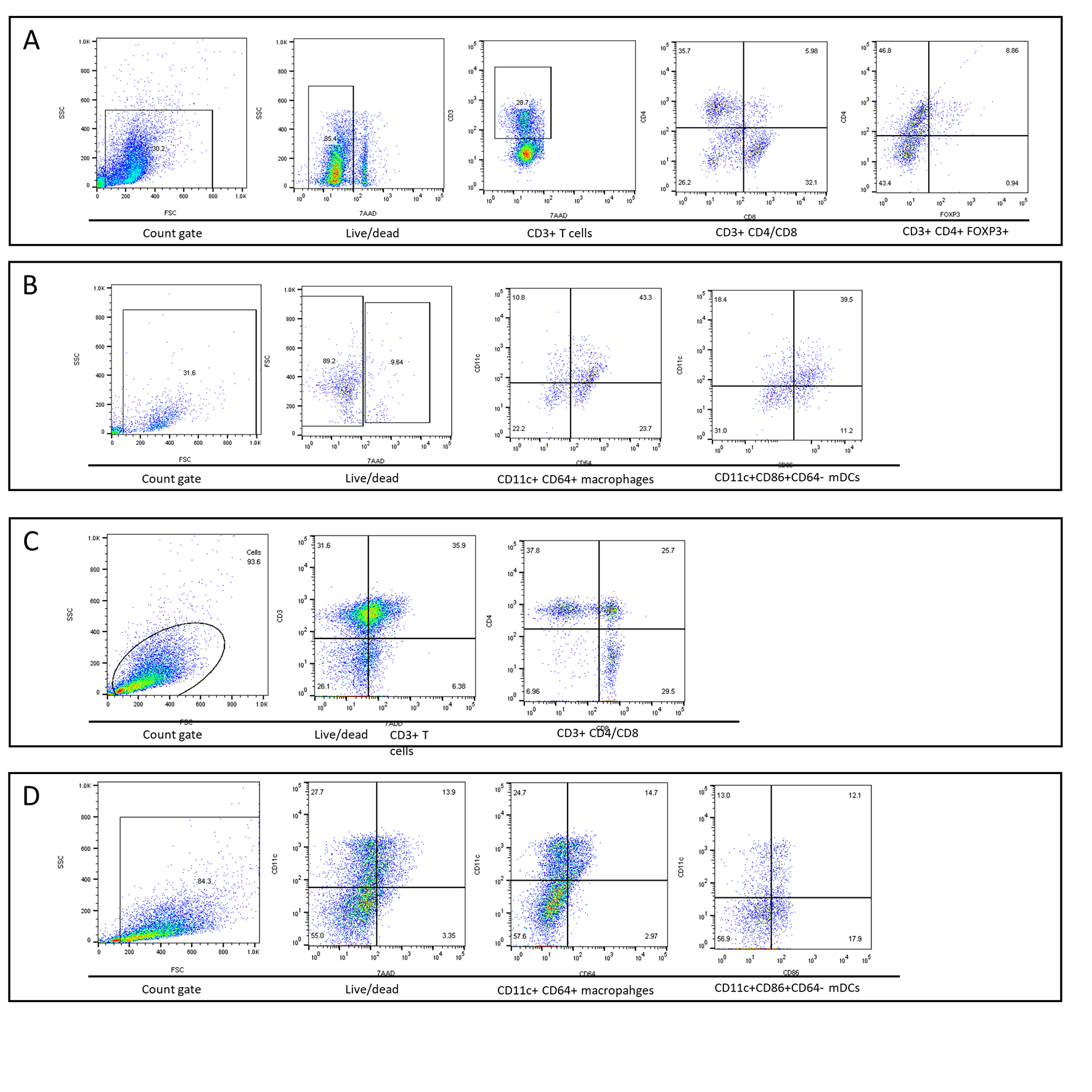

Supplement: Supplementary Figure 1 — Gating scheme for all cells. (A) Gating scheme for T-cells in tumor. CD45+ cells from tumor were gated on FSC/SSC, then live vs. dead, then CD3+. CD3+ T-cells were gated as CD4+. CD8+ or CD4+ FOXP3+ as shown. (B) Gating scheme for dendritic cells and macrophages in tumor. CD45+ cells from tumor were gated on FSC/SSC, then live vs. dead, then CD11c+. Macrophages were gated as CD64+, mDCs as CD64- CD86. (C) Gating scheme for T-cells in spleen. CD45+ cells spleen was gated on FSC/SSC, then live vs. dead, then CD3+. CD3+ T-cells were gated as CD4+. CD8+ or CD4+ as shown. (D) Gating scheme for dendritic cells and macrophages in spleen. CD45+ cells from spleen were gated on FSC/SSC, then live vs. dead, then CD11c+. Macrophages were gated as CD64+, mDCs as CD64- CD86. [file Image_1.TIF]

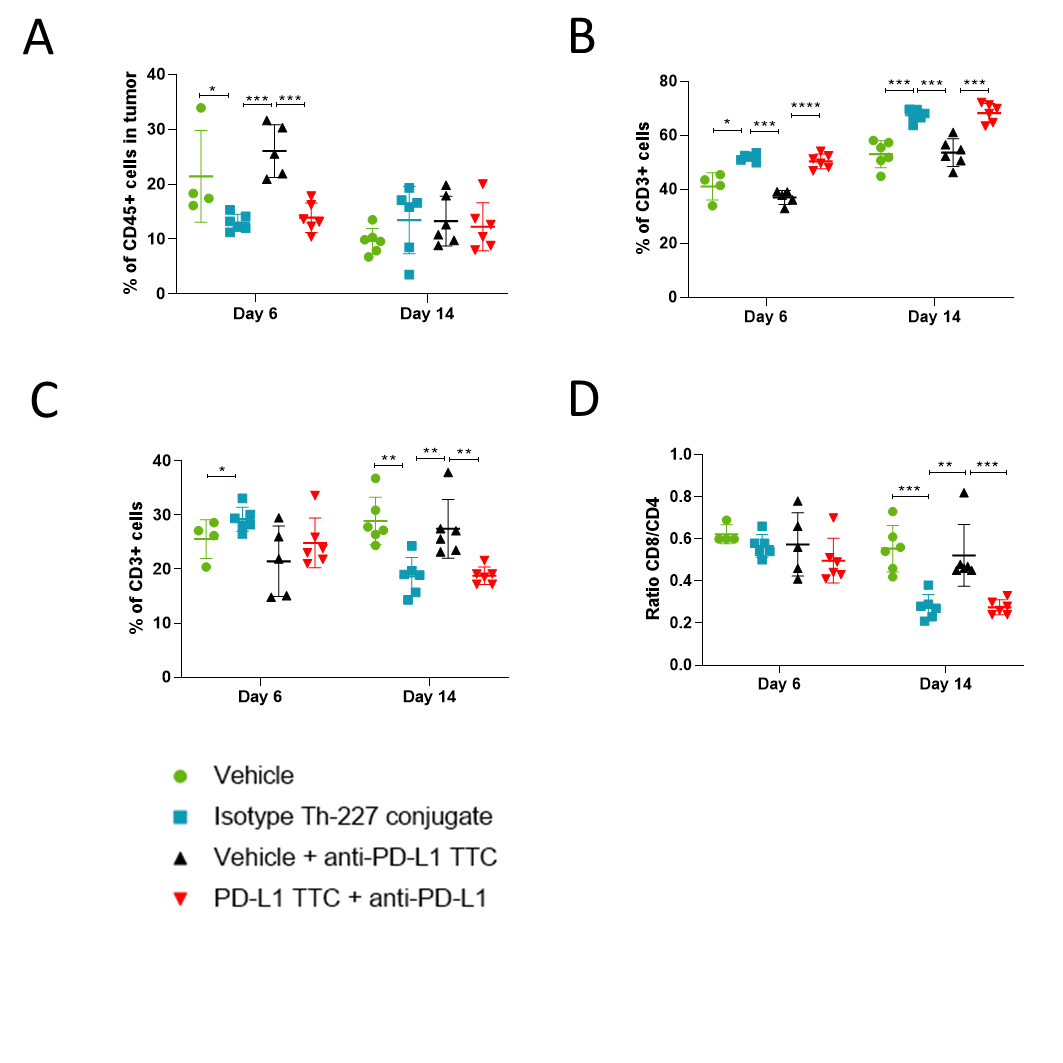

Supplement: Supplementary Figure 2 — (A) Lymphocytes (%) that are CD3+ in spleen, in each treatment group, n = 5/group. (B) CD3+ cells (%) that are CD4+ CD8- in spleen, n = 5/group. (C) CD3+ cells (%) that are CD8+ CD4- in spleen, n = 5/group. (D) Ratio of CD8+ cells to CD4+ in spleen, n = 5/group. Student’s t-test *p < 0.05, **p < 0.01, ***p < 0.001. [file Image_2.TIF]

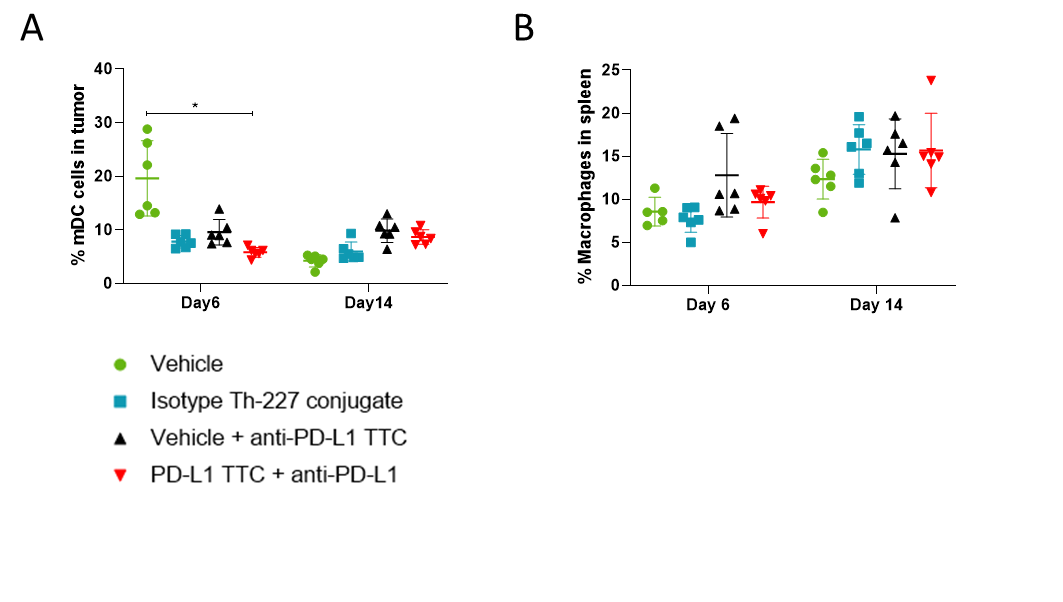

Supplement: Supplementary Figure 3 — (A) Mature DCs [CD11c+, CD86+, CD64-(%)] in spleen, in each treatment group, n = 5/group. (B) Macrophages [CD11c+, CD64+ (%)] in spleens, in each treatment group, n = 5/group. Student’s t-test *p < 0.05. [file Image_3.TIF]

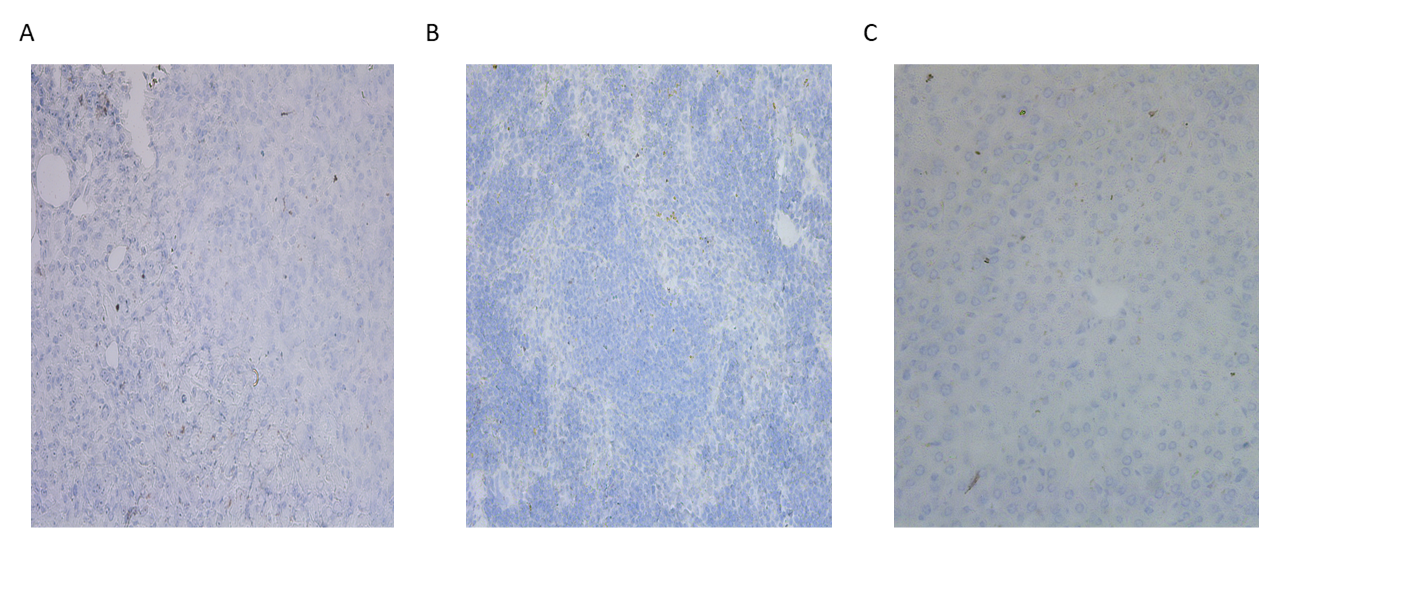

Supplement: Supplementary Figure 4 — Blank IHC samples (A) Blank MC-38 tumor. (B) Blank C57BL/6 spleen. (C) Blank C57BL/6 liver. [file Image_4.TIF]
